# Uncovering Differential Item Functioning effects using MIMIC and mediated MIMIC models

**DOI:** 10.3389/fpsyg.2023.1268074

**Published:** 2023-10-23

**Authors:** Ioannis Tsaousis, Maisaa Taleb S. Alahmandi, Halimah Asiri

**Affiliations:** ^1^Department of Psychology, National and Kapodistrian University of Athens (NKUA), Athens, Greece; ^2^Education and Training Evaluation Commission (ETEC), Riyadh, Saudi Arabia

**Keywords:** Differential Item Functioning (DIF), uniform DIF, MIMIC approach, mediation analysis, mediated MIMIC model

## Abstract

The aim of this study was twofold: first, to examine the presence of bias across gender in a scholastic achievement test named the Academic Achievement Test (AAT) for the Science Track. Second, to understand the underlying mechanism that causes these bias effects by examining the effect of general cognitive ability as a mediator. The sample consisted of 1,300 Saudi high school students randomly selected from a larger pool of 173,133 participants to reduce the effects of excessive power. To examine both goals, the Multiple Indicators Multiple Causes (MIMIC) approach for detecting Differential Item Functioning (DIF) items was used. The results showed that 13 AAT items exhibited DIF effects for different gender groups. In most of these items, male participants were more likely to answer them correctly than their female counterparts. Next, the mediated MIMIC approach was applied to explore possible underlying mechanisms that explain these DIF effects. The results from this study showed that general cognitive ability (i.e., General Aptitude Test - GAT) seems to be a factor that could explain why an AAT item exhibits DIF across gender. It was found that GAT scores fully explain the DIF effect in two AAT items (full mediation). In most other cases, GAT helps account for only a proportion of the DIF effect (partial mediation). The results from this study will help experts improve the quality of their instruments by identifying DIF items and deciding how to revise them, considering the mediator’s effect on participants’ responses.

## 1. Introduction

In modern psychometrics, there is an increasing interest in identifying and understanding what causes a Differential Item Functioning (DIF) effect (Raykov and Marcoulides, 2011). DIF refers to a situation where an item performs differently across groups of individuals even though those individuals are supposed to have the same level of the trait being measured (Dorans and Holland, 1993). DIF can be caused by cultural, societal, or demographic variables, and it can undermine the fairness and validity of a test or assessment (Ackerman, 1994). DIF can be categorized into two main types: uniform and non-uniform. An item shows uniform DIF when the performance of one group is always superior to another group for each ability level. On the other hand, non-uniform DIF occurs when an item’s bias varies across different levels of the latent trait. Therefore, it is important first to identify DIF items and remove them from the scale.

Several statistical methods for identifying items with DIF have been proposed within the Classical Test Theory (CTT) and the Item Response Theory (IRT). Within the IRT framework, the model-based likelihood ratio test is an approach that is typically used to evaluate the significance of observed differences in parameter estimates between groups (Thissen et al., 1993). Other methods include the likelihood ratio goodness-of-fit test (Thissen et al., 1986) and the simultaneous item bias test (SIBTEST) method (Shealy and Stout, 1993). Within the CTT framework, the Mantel–Haenszel (MH) approach (Holland and Thayer, 1988) and the logistic regression (LR) procedure (Swaminathan and Rogers, 1990) are some of the most popular approaches.

**Figure 1:**
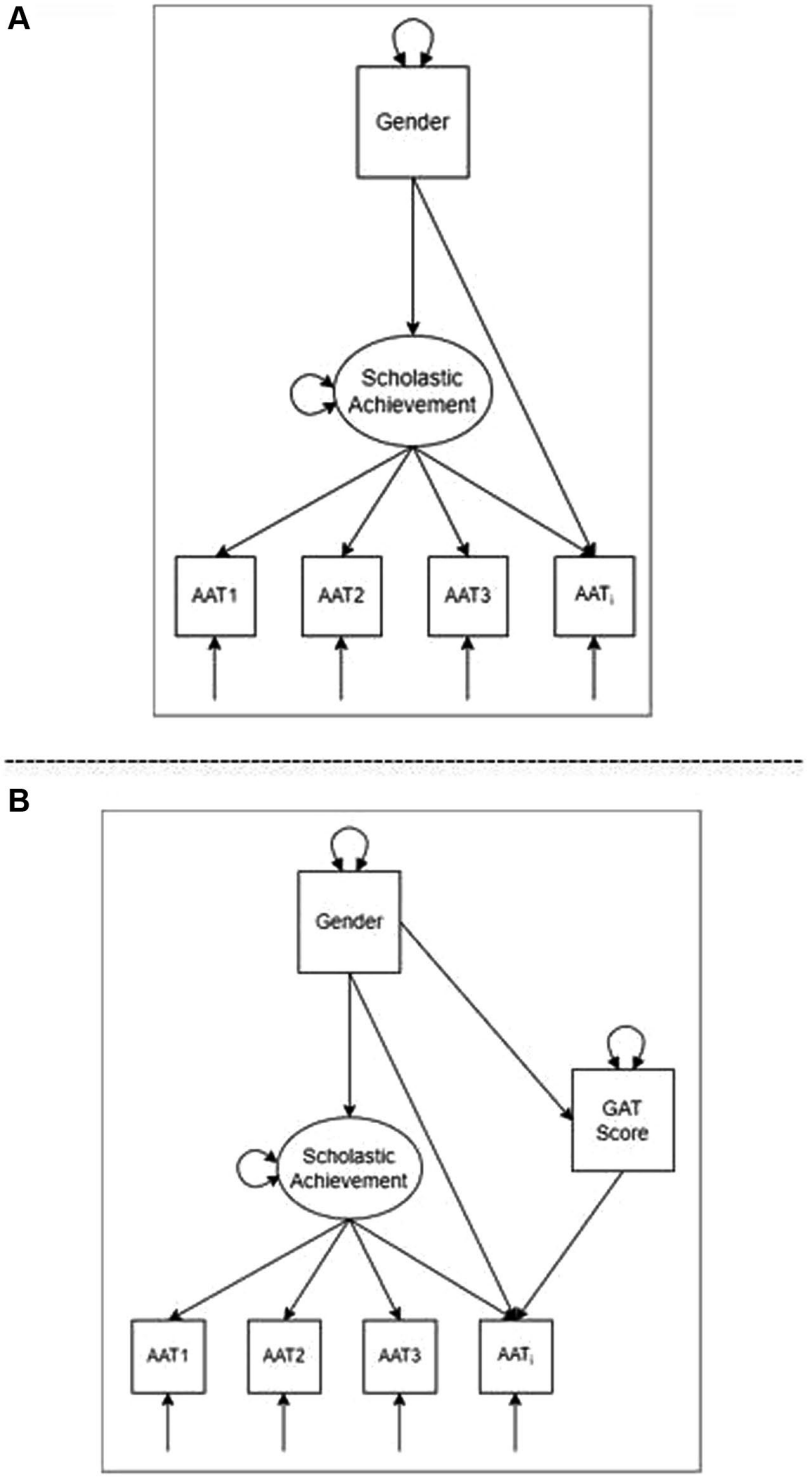
MIMIC and mediated MIMIC models for testing DIF effects. **(A)** The standard MIMIC approach to detecting DIF. **(B)** The mediated MIMIC approach to detecting DIF.

Structural Equation Modelling (SEM) also provides a comprehensive framework for examining and understanding the DIF issue (Camilli and Shepard, 1994). Within this context, several different methods have been suggested, including the Multi-Group CFA method (MG-CFA; Pae and Park, 2006), the modification indices method (Chan, 2000), and the Multiple-Indicator, Multiple-Causes approach (MIMIC; MacIntosh and Hashim, 2003). One of the major advantages of the MIMIC approach over the MG-CFA method is that it uses the entire sample of responses to estimate model parameters and test for DIF (Chun et al., 2016). In this case, the total sample size needed for detecting DIF is smaller than that needed in the MG-CFA approach, where model parameters are estimated separately for each contrasted group (Muthén, 1989). Additionally, several explanatory variables (e.g., demographic) can be included within a MIMIC model, allowing us to identify possible causes of DIF. An example of a MIMIC DIF model is shown in Figure 1 (upper panel), in which a grouping variable (Gender) has direct effects on the items of the scale (e.g., AAT_i_) and the latent mean (e.g., scholastic achievement) simultaneously.

Recently, Cheng et al. (2016) proposed a method for detecting DIF items in which they combined the MIMIC methodology with mediation analysis to uncover possible causes of DIF effects. In mediation analysis, it is hypothesized that the independent variable (e.g., Gender) affects the dependent variable (e.g., the item AAT_i_) *via* an intervening variable called the mediator (e.g., GAT Score) (Baron and Kenny, 1986). The effect of the mediator in the relationship between the independent and dependent variables can be either full (the direct relationship between Gender and AAT_i_ disappears after the effect of the mediator is controlled) or partial (the mediator can only explain a part of the relationship between the Gender and AAT_i_). This relationship constitutes a uniform DIF and is graphically presented in Figure 1 (lower panel).

## 2. Research purpose and specific aims

Previous studies have shown that gender is assumed to considerably affect students’ academic performance since many studies have shown that boys and girls perform differently (e.g., Voyer and Voyer, 2014). Nevertheless, not all studies agree on the direction and magnitude of this difference (e.g., Else-Quest et al., 2010), and the gender gap in academic attainment is still an open question. This study uses gender as a grouping variable to examine possible DIF effects on academic achievement. It was hypothesized that the response to an AAT item (e.g., AAT_i_), which measures scholastic achievement (i.e., the latent variable), involves some general cognitive ability level (i.e., the mediator). Thus, cognitive ability, as measured by the General Aptitude Test (GAT), will completely or partially mediate the relationship between gender and a response to an AAT item when controlling for scholastic achievement. In this study, only uniform DIF was examined.

## 3. Methods

### 3.1. Participants and procedure

Previous simulation studies on Differential Item Functioning (DIF) and mediation analysis suggested that with a sample size as large as 1,000 or up and a mediation effect of 0.10 or up, the analysis has enough power to provide robust results (Cheng et al., 2016). Therefore, to reduce the effects of excessive power, a sample of 1,300 participants was randomly selected from a larger pool of 173,133 high school students who completed an achievement test as part of a national examination process. Of them, 648 (49.8%) were males, and 652 (50.2%) were females. The participants’ mean age was 17.99 (SD = 0.53). In terms of place of residence, participants originated from all 13 regions of Saudi Arabia. The study was conducted in accordance with the Declaration of Helsinki and approved by the Institutional Review Board (or Ethics Committee) of the Education & Training Evaluation Commission (Approval Code: TR369-2023, Approval Date: 20/11/2022).

### 3.2. Measures

#### 3.2.1. The academic achievement test for the science track (AAT; education and training evaluation commission - ETEC)

The AAT is a 44-item admission test that measures achievement level in accordance with university study readiness standards. It consists of four subscales that focus on the general outcomes of the following courses: First, second-, and third-year Biology (12 items), Chemistry (10 items), Physics (10 items), and Mathematics (12 items) of the secondary stage (grades 10, 11, and 12). The AAT test items are in a multiple-choice format and are scored as correct (1) or wrong (0). The test has a 50-min duration and is presented in Arabic.

#### 3.2.2. General aptitude test (GAT) for science major (education and training evaluation commission - ETEC)

This is a general cognitive ability test developed in the Arabic language that measures analytical and deductive skills. It is composed of two cognitive domains: (a) language-related skills (68 items) and (b) numerical-related skills (52 items). Each domain comprises several subdomains, including word meaning, sentence completion, reading comprehension, arithmetic, analysis, geometry, etc. The global cognitive ability factor composed of the scores from the two domain scales was the only available score from this test in this study. All scores were transformed into standard scores (T-scores), with a range of 0–100.

### 3.3. Data analysis

Before examining DIF effects and possible causes within the Structural Equation Modeling (SEM) framework, the measurement model specification of each of the four AAT scales was examined. The following goodness of fit indices were used: the Comparative Fit Index (CFI), the Tucker-Lewis Index (TLI), the Root Mean Square Error of Approximation (RMSEA), and the Standardized Root Mean Square Residual (SRMR). CFI and TLI values higher than 0.90 indicate an acceptable fit (with values >0.95 being ideal), and RMSEA and SRMR values up to 0.08 indicate a reasonable fit (with values <0.05 indicating an excellent fit (Hu and Bentler, 1999).

Next, the MIMIC model approach was used to detect DIF items across the different AAT scales. The *MIMIC model with scale purification* (*M-SP*) method was used (Wang and Shih, 2010) for each scale separately. In this approach, the direct effect of the grouping variable (e.g., gender) on an item response (e.g., AAT*_i_*) is estimated. In Figure 1 (upper panel), this relationship is represented by a direct path from Gender to item AAT*_i_*. The direct effect represents the difference in item response between the two levels of the grouping variable (i.e., males vs. females) given the same scholastic achievement ability (latent variable). If the direct effect is significant, this indicates a DIF effect. The indirect effect is represented by a path from grouping variable to latent variable and indicates whether the mean of the latent variable across groups is different. The same procedure will be followed for all AAT items, one at a time. It should also be noted that Bonferroni correction will be adopted to control for the Type I error (Dunn, 1961).

After identifying DIF items, the mediated MIMIC approach was used to uncover possible causes of the emerging DIF effects. As discussed earlier, a mediator (e.g., GAT score) can mediate the relationship between group membership (e.g., gender) and an item response (AAT*_i_*), conditioning on the latent trait (e.g., scholastic achievement). Therefore, when we fit a DIF item (found in the previous analysis step) in the mediation model, we obtain direct and indirect effects for each model. If the direct effect (from the grouping variable to the item) becomes non-significant when the mediator (i.e., GAT score) is taken into account in this relationship (from the grouping variable to the mediator and then to the item), we have full mediation (the indirect effect is significant). This means that the mediator fully explains the DIF effect. On the other hand, if the direct effect is still significant when the mediator is entered into the equation, and the indirect effect is significant, we have partial mediation. In this case, the mediator explains to some extent the DIF effect, but maybe additional mediators are needed to explain the causes of the DIF effect fully. All analyses were conducted using Mplus 8.03 (Muthén and Muthén, 1998-2018).

## 4. Results

First, the measurement model of each AAT scale (i.e., Biology, Chemistry, Physics, and Mathematics) was examined *via* CFA. A unidimensional structure for each scale was hypothesized. In Table 1, the results from the CFA are reported. The results showed that all measurement models fit the data very well.

**Table 1:** Model fit indices for AAT scales.

Scales	χ^2^	*df*	CFI	TLI	RMSEA (95% CIs)	SRMR
Biology	79.610^*^	54	0.973	0.967	0.019 (0.009–0.028)	0.038
Chemistry	77.599^**^	35	0.984	0.980	0.031 (0.021–0.040)	0.043
Physics	111.354^**^	35	0.924	0.903	0.041 (0.033–0.050)	0.057
Mathematics	84.707^**^	54	0.985	0.981	0.021 (0.012–0.029)	0.037

*χ*^2^, chi-square goodness of fit statistic; df, degrees of freedom; CFI, Comparative Fit Index; TLI, Tucker Lewis Index; RMSEA, Root Mean Square Error of Approximation; 95% CIs = 95% Confidence Intervals; SRMR, Standardized Root Mean Square Residual. ^**^ Models are significant at *p* < 0.001; ^*^ Models are significant at *p* < 0.01.

Next, a MIMIC approach was applied to detecting uniform DIF items across gender for all AAT scales. During the process of identifying DIF items, every item within each scale was regressed on the grouping variable, with all other items presumed as non-DIF items and serving as the anchor set. In the grouping variable (i.e., gender), males were coded as 0 (the reference group) and females as 1 (the focal group). A negative z value indicates that males at the same level of scholastic achievement as females are more likely to respond to the item correctly. To identify potential DIF items, the following equation was applied:

$$Y_{ij}=\lambda_{j}*\theta_{i}+\beta_{j}z_{i}+e_{ij}$$

Where,

Υ*_ij_* = the latent response for item *j* for participant *i.*

λ*_j_* = the factor loading of item *j.*

θ*_i_* = the latent ability of the participant *i.*

z*_i_* = the grouping indicator of the participant *i.*

β*_j_* = the regression coefficient of the corresponding grouping variable, and.

e*_ij_* = the random error term.

If β*_j_* is non-significant, then item *j* is the same across groups of variable z*_i_*. However, if β*_j_* is significant, it designates a difference in the response probabilities across groups of variable z*_i_*, designating a DIF item. Practically, DIF is detected when the direct relationship between the group variable (gender) and the item in question is statistically significant. It should be noted that the Benjamini-Hochberg correction was applied to control for false discovery rate (Benjamini and Hochberg, 1995). Table 2 presents the results from the DIF analysis.

**Table 2:** MIMIC examination for DIF across gender.

Items	Estimate (β)	S.E.	*z* value	*p*-value
Biology scale				
Bio1	−0.044	0.036	−1.226	ns
Bio2	−0.008	0.032	−0.255	ns
Bio3	0.042	0.031	1.333	ns
Bio4	0.028	0.032	0.867	ns
Bio5	−0.055	0.032	−1.697	ns
Bio6	−0.013	0.033	−0.405	ns
Bio7	−0.099	0.034	−2.888	0.004
Bio8	0.095	0.031	3.027	0.002
Bio9	0.064	0.032	2.015	ns
Bio10	−0.088	0.037	−2.393	ns
Bio11	0.034	0.032	1.059	ns
Bio12	0.031	0.031	0.974	ns
Chemistry scale				
Chem13	0.116	0.036	3.214	0.001
Chem14	−0.012	0.033	−0.372	ns
Chem15	−0.121	0.037	−3.316	0.001
Chem16	0.050	0.038	1.322	ns
Chem17	0.046	0.031	1.481	ns
Chem18	−0.100	0.034	−2.910	0.004
Chem19	0.065	0.031	2.0101	ns
Chem20	−0.080	0.032	−2.456	0.014
Chem21	−0.022	0.033	−0.668	ns
Chem22	0.023	0.034	0.067	ns
Physics scale				
Phys23	0.056	0.034	1.638	ns
Phys24	0.018	0.032	0.570	ns
Phys25	−0.166	0.040	−4.114	0.001
Phys26	−0.177	0.041	−4.330	0.001
Phys27	−0.083	0.045	−1.845	ns
Phys28	−0.117	0.037	−3.199	0.001
Phys29	0.140	0.032	4.409	0.001
Phys30	−0.048	0.035	−1.371	ns
Phys31	0.186	0.031	5.921	0.001
Phys32	−0.063	0.037	−1.689	ns
Mathematics scale				
Math33	0.023	0.033	0.0700	ns
Math34	−0.128	0.031	−4.143	0.001
Math35	−0.068	0.032	−2.146	ns
Math36	0.077	0.032	2.391	ns
Math37	−0.042	0.032	−1.319	ns
Math38	−0.008	0.032	−0.258	ns
Math39	−0.023	0.032	−0.718	ns
Math40	−0.029	0.032	−0.913	ns
Math41	0.052	0.031	1.706	ns
Math42	0.023	0.041	0.552	ns
Math43	0.085	0.030	2.784	0.005
Math44	0.012	0.033	0.375	ns

Bio, Biology; Chem, Chemistry; Phys, Physics; Math, Mathematics.

The analysis uncovered 13 DIF items. For example, in the Biology scale, items 7 and 8 were detected as DIF items. In item 7, the z value (−2.888) indicates that controlling for scholastic achievement, a male participant is more likely to respond correctly than a female participant. In item 8, on the other hand, the positive z value indicates that female participants are more likely to respond correctly than male participants, although they are at the same level of scholastic achievement.

After this step, the mediated MIMIC approach was applied in an attempt to understand what causes DIF in these items. It was hypothesized that general cognitive ability (i.e., GAT) could be a mediator that mediates the relationship between the grouping variable and the response to a specific item. Table 3 presents the results of the mediation analysis within a MIMIC model.

**Table 3:** Direct and indirect (mediation) effects for DIF items.

Item	Direct effect	*p*-value	Indirect effect	*p*-value
Bio7	−0.107	0.002	0.019	0.006
Bio8	0.090	0.005	0.022	0.001
Chem 13	0.113	0.003	0.039	0.001
Chem 15	−0.092	0.017	0.026	0.004
Chem 18	−0.056	0.121	0.040	0.001
Chem 20	−0.048	0.155	0.033	0.001
Phys25	−0.166	0.001	0.023	0.003
Phys26	−0.178	0.001	−0.003	0.595
Phys28	−0.114	0.002	0.036	0.001
Phys29	0.142	0.001	0.029	0.001
Phys31	0.190	0.001	0.020	0.001
Math34	−0.136	0.001	0.021	0.002
Math43	0.081	0.010	0.025	0.001

Bio, Biology; Chem, Chemistry; Phys, Physics; Math, Mathematics.

The results showed that cognitive ability seems to be a factor that could explain why an AAT item exhibits DIF across gender. GAT fully explains the DIF effect in two AAT items (i.e., Chem18 and Chem20) since the direct effect is no longer significant after the mediator enters the equation (full mediation). In both cases, the effect of the GAT score on the probability of correct response is positive (a_7_ = 0.323, SE = 0.048, z = 6.723, *p* = 0.001, and a_8_ = 0.265, SE = 0.034, z = 6.074, p = 0.001, respectively). This means that the higher the GAT score, the higher the probability of answering the item correctly. However, the direct effect on both items is negative (β_7_ = −0.056, SE = 0.036, *p* = 0.121, and β_8_ = −0.048, SE = 0.034, *p* = 0.155). This finding suggests that females with the same GAT score are less likely to answer this item correctly compared to males.

In most other cases, GAT helps account for only a proportion of the DIF effect (partial mediation). Obviously, additional factors intervene in the relationship between gender and answering an item correctly and cause DIF effects. Only in one case (i.e., Phys26) could GAT not explain why male participants are more likely to respond correctly to this item than female participants, although both are at the same underlying level of cognitive ability. Interestingly, males were more likely to respond correctly to some items than females (i.e., Bio7, Chem15, Chem18, Chem20, Phys28, and Math34). But when the GAT score was taken into account (i.e., as a mediator), the probability of correctly answering these items was higher for females than for males.

## 5. Discussion

The aim of this study was twofold: first, to examine whether there are gender differences in the probability of correctly answering an item of the AAT. In other words, whether there are DIF items in terms of gender. Second, to understand the underlying mechanism that causes these DIF effects. The first aim, detecting DIF items, was examined *via* a MIMIC approach. MIMIC models have been used extensively for identifying items with DIF (Muthèn, 1985) since it has been found that they work equally well with other methods (Woods, 2009). This study used a MIMIC model to detect possible DIF items across gender for a scholastic achievement test (i.e., AAT). The analysis revealed that 13 AAT items exhibited DIF across gender (i.e., two from the Biology scale, four from the Chemistry scale, five from the Physics scale, and two from the Mathematics scale). Furthermore, in most (9 out of 13), male participants were more likely to answer the items correctly than their female counterparts.

The second aim of this study, to uncover possible causes of DIF, was examined *via* the mediated MIMIC approach. Mediation analysis is a statistical method that provides a framework for understanding why certain phenomena in the relationship among variables occur. Using this analysis within a MIMIC model for detecting DIF, we can explore possible underlying mechanisms that explain these DIF effects. It was hypothesized that general cognitive ability, as measured by the General Aptitude Test (GAT), could mediate the relationship between the grouping variable (e.g., gender) and the response to a specific item. If a mediation effect exists, we can explain why a DIF effect occurs, depending on the Type of mediation (full or partial).

The results from this study showed that general cognitive ability fully explains the DIF effect in two AAT items (i.e., Chem18 and Chem20). In most other cases, GAT helps account for only a proportion of the DIF effect (partial mediation). It seems that additional factors intervene in the relationship between gender and answering an item correctly and cause DIF effects. Interestingly, from all detected DIF items, only for one item (Phys26), GAT could not explain why the DIF effect occurred.

This study offers valuable information regarding DIF effects and the possible causes of these effects. Using the MIMIC approach, DIF effects were examined within the mediation analysis framework. As a result, it was revealed that general cognitive ability mediates the relationship between gender and the probability of success in an item and provides a context for understanding the underlying mechanism of why DIF effects occurred. Therefore, this study will help experts improve the quality of their instruments by identifying DIF items and deciding how to revise them, considering the mediator’s effect on participants’ responses. Taking the Biology scale as an example, when Subject Matter Experts (SMEs) are asked to generate items, they should pay careful attention to producing items that are purely related to specific knowledge (i.e., physics) rather than general cognitive ability.

The present study also has certain limitations. First, only GAT scores were available as potential mediators. Future studies should explore the role of other variables, including cognitive (e.g., GPA) and emotional (e.g., self-efficacy) constructs, that could be used to explain the emergence of DIF effects. Second, only gender was examined as a potential grouping variable. In future studies, additional variables (e.g., Type of school: public vs. private) could be examined as potential causes of DIF. Finally, in this study, only uniform DIF was investigated. We would like to expand this approach to examine also non-uniform DIF effects. This type of DIF examines whether an item discriminates differently between the groups in question. Thus, important information about non-uniform DIF effects could be revealed by conceptualizing DIF within the context of moderated mediation analysis (Montoya and Jeon, 2020).

## Data availability statement

The data analyzed in this study is subject to the following licenses/restrictions: The data that supports the findings of this study are available from the Education and Training Evaluation Commission (ETEC). Restrictions apply to the availability of these data, which were used under license for this study. Data are available from the authors upon reasonable request and with the permission of the ETEC. Requests to access these datasets should be directed to MA, m.ahmadi@etec.gov.sa.

## Author contributions

IT: Conceptualization, Formal analysis, Methodology, Writing – original draft, Writing – review & editing. MA: Methodology, Writing – review & editing. HA: Data curation, Writing – review & editing.

## References

[ref1] Ackerman T. A. Evans J. A. (1994). The Influence of Conditioning Scores In Performing DIF Analyses. Applied Psychological Measurement 18, 329–342.

[ref2] Baron R. M. Kenny D. A. (1986). The moderator–mediator variable distinction in social psychological research: conceptual, strategic, and statistical considerations. J. Pers. Soc. Psychol. 51, 1173–1182. doi: 10.1037/0022-3514.51.6.1173 3806354

[ref3] Benjamini Y. Hochberg Y. (1995). Controlling the false discovery rate: a practical and powerful approach to multiple testing. J. R. Stat. Soc. Ser. B 57, 289–300. doi: 10.1111/j.2517-6161.1995.tb02031.x

[ref5] Camilli G. Shepard L. A. (1994). Methods for identifying biased test items. London: Sage.

[ref6] Chan D. (2000). Detection of differential item functioning on the Kirton adaption-innovation inventory using multiple-group mean and covariance structure analyses. Multivar. Behav. Res. 35, 169–199. doi: 10.1207/S15327906MBR3502_2, PMID: 26754082

[ref7] Cheng Y. Shao C. Lathrop Q. N. (2016). The mediated MIMIC model for understanding the underlying mechanism of DIF. Educ. Psychol. Meas. 76, 43–63. doi: 10.1177/0013164415576187, PMID: 29795856 PMC5965572

[ref8] Chun S. Stark S. Kim E. S. Chernyshenko O. S. (2016). MIMIC methods for detecting DIF among multiple groups: exploring a new sequential-free baseline procedure. Appl. Psychol. Meas. 40, 486–499. doi: 10.1177/0146621616659738, PMID: 29881065 PMC5978634

[ref10] Dorans N. J. Holland P. W. (1993). “DIF detection and description: mantel-Haenszel and standardization” in Differential item functioning. eds. Holland P. W. Wainer H. (Hillsdale, NJ: Lawrence Erlbaum), 35–66.

[ref11] Dunn J. O. (1961). Multiple comparisons among means. J. Am. Stat. Assoc. 56, 52–64. doi: 10.1080/01621459.1961.10482090

[ref12] Else-Quest N. M. Hyde J. S. Linn M. C. (2010). Cross-national patterns of gender differences in mathematics: a meta-analysis. Psychol. Bull. 136, 103–127. doi: 10.1037/a0018053, PMID: 20063928

[ref13] Holland P. W. Thayer D. T. (1988). “Differential item performance and the mantel-Haenszel procedure” in Test validity. eds. Wainer H. Braun H. I. (Hillsdale, NJ: Lawrence Erlbaum), 129–145.

[ref14] Hu L. T. Bentler P. M. (1999). Cut-off criteria for fit indexes in covariance structure analysis: conventional criteria versus new alternatives. Struct. Equ. Model. 6, 1–55. doi: 10.1080/10705519909540118

[ref15] MacIntosh R. Hashim S. (2003). Variance estimation for converting MIMIC model parameters to IRT parameters in DIF analysis. Appl. Psychol. Meas. 27, 372–379. doi: 10.1177/0146621603256021

[ref001] Montoya A. K. Jeon M. (2020). MIMIC Models for Uniform and Nonuniform DIF as Moderated Mediation Models. Applied psychological measurement 44, 118–136. 32076356 10.1177/0146621619835496 PMC7003182

[ref002] Muthén B. O. (1989). Latent variable modeling in heterogeneous populations. Psychometrika 54, 557–585.

[ref16] Muthèn B. O. (1985). A method for studying the homogeneity of test items with respect to other relevant variables. J. Educ. Stat. 10, 121–132. doi: 10.3102/10769986010002121

[ref17] Muthén L. K. Muthén B. O. (1998-2018). Mplus User’s Guide. 8th Edn. Los Angeles, CA: Muthén & Muthén.

[ref18] Pae T. I. Park G. P. (2006). Examining the relationship between differential item functioning and differential test functioning. Lang. Test. 23, 475–496. doi: 10.1191/0265532206lt338oa

[ref19] Raykov T. Marcoulides G. A. (2011). Introduction to psychometric theory Routledge.

[ref20] Shealy R. T. Stout W. F. (1993). “An item response theory model for test bias and differential item functioning” in Differential item functioning. eds. Holland P. W. Wainer H. (Hillsdale, NJ: Lawrence Erlbaum Associates), 197–240.

[ref21] Swaminathan H. Rogers H. J. (1990). Detecting item bias using logistic regression procedures. J. Educ. Meas. 27, 361–370. doi: 10.1111/j.1745-3984.1990.tb00754.x

[ref22] Thissen D. Steinberg L. Gerrard M. (1986). Beyond group-mean differences: the concept of item bias. Psychol. Bull. 99, 118–128. doi: 10.1037/0033-2909.99.1.118

[ref23] Thissen D. Steinberg L. Wainer H. (1993). “Detection of differential item functioning using the parameters of item response models” in Differential item functioning. eds. Holland P. W. Wainer H. (Hillsdale, NJ: Lawrence Erlbaum Associates), 67–113.

[ref003] Voyer D. Voyer S. D. (2014). Gender differences in scholastic achievement: a meta-analysis. Psychological Bulletin 140, 1174–1204. doi: 10.1037/a0036620 24773502

[ref24] Wang W. C. Shih C. L. (2010). MIMIC methods for assessing differential item functioning in polytomous items. Appl. Psychol. Meas. 34, 166–180. doi: 10.1177/0146621609355279

[ref25] Woods C. M. (2009). Evaluation of MIMIC-model methods for DIF testing with comparison to two-group analysis. Multivar. Behav. Res. 44, 1–27. doi: 10.1080/00273170802620121, PMID: 26795105

