# Supplementary material for: Uncovering Differential Item Functioning effects using MIMIC and mediated MIMIC models
Source: Front Psychol. 2023 Oct 23;14:1268074. doi: 10.3389/fpsyg.2023.1268074 (PMC10626479; doi:10.3389/fpsyg.2023.1268074)
Supplement: Supplementary file 1 [file Table_1.DOCX]

Mplus Syntax Codes for MIMIC DIF and Mediated MIMIC DIF Models

MIMIC model for DIF detection

title: Uniform DIF - Chemistry (AAT18)

data: file is DIF data1300.dat;

variable: names are gender AAT1-AAT44 GAT;

usevariables are gender AAT13 AAT14 AAT15 AAT16 AAT17 AAT18 AAT19 AAT20 AAT21 AAT22;

categorical are AAT13 AAT14 AAT15 AAT16 AAT17 AAT18 AAT19 AAT20 AAT21 AAT22;

analysis:

estimator is MLR;

model:

Chem by AAT13 AAT14 AAT15 AAT16 AAT17 AAT18 AAT19 AAT20 AAT21 AAT22*;

Chem@1;

Chem on gender;

AAT18 on gender;

output: sampstat stdyx;

Mediated MIMIC model for DIF detection

title: Uniform DIF with GAT - Chemistry (AAT18)

data: file is DIF data1300.dat;

variable: names are gender AAT1-AAT44 GAT;

usevariables are gender AAT13 AAT14 AAT15 AAT16 AAT17 AAT18 AAT19 AAT20 AAT21 AAT22 GAT;

categorical are AAT13 AAT14 AAT15 AAT16 AAT17 AAT18 AAT19 AAT20 AAT21 AAT22;

analysis:

estimator is MLR;

model:

Chem by AAT13 AAT14 AAT15 AAT16 AAT17 AAT18 AAT19 AAT20 AAT21 AAT22*;

Chem on gender;

AAT18 on GAT;

GAT on gender;

AAT18 on gender;

MODEL INDIRECT:

AAT18 ind gender;

output: sampstat stdyx;
